# Effects of restricting pub closing times on night-time assaults in an Australian city

**DOI:** 10.1111/j.1360-0443.2010.03125.x

**Published:** 2011-02

**Authors:** Kypros Kypri, Craig Jones, Patrick McElduff, Daniel Barker

**Affiliations:** 1 School of Medicine and Public Health, University of NewcastleNSW, Australia; 2 NSW Bureau of Crime Statistics and ResearchNSW, Australia

**Keywords:** Alcohol, assault, closing, hotels, licensed premises, opening hours, pubs, trading hours

## Abstract

**Aims:**

In March 2008 the New South Wales judiciary restricted pub closing times to 3 a.m., and later 3.30 a.m., in the central business district (CBD) of Newcastle, Australia. We sought to determine whether the restriction reduced the incidence of assault.

**Design:**

Non-equivalent control group design with before and after observations.

**Setting:**

Newcastle, a city of 530 000 people.

**Participants:**

People apprehended for assault in the CBD and nearby Hamilton, an area with a similar night-time economy but where no restriction was imposed.

**Measurements:**

Police-recorded assaults in the CBD before and after the restriction were compared with those in Hamilton. Cases were assaults occurring from 10 p.m.–6 a.m. from January 2001–March 2008, with April 2008–September 2009 as the post-restriction period. We also examined changes in assault incidence by time of night. Negative binomial regression with time, area, time × area interaction terms and terms for secular trend and seasonal effects was used to analyse the data. Autocorrelation was examined using generalized estimating equations.

**Findings:**

In the CBD, recorded assaults fell from 99.0 per quarter before the restriction to 67.7 per quarter afterward [incidence rate ratio (IRR): 0.66, 95% confidence interval (CI): 0.55–0.80]. In the same periods in Hamilton, assault rates were 23.4 and 25.5 per quarter, respectively (IRR: 1.02, 95% CI: 0.79–1.31). The relative reduction attributable to the intervention was 37% (IRR = 0.63, 95% CI: 0.47–0.81) and approximately 33 assault incidents were prevented per quarter.

**Conclusion:**

This study indicates that a restriction in pub closing times to 3/3.30 a.m. in Newcastle, NSW, produced a large relative reduction in assault incidence of 37% in comparison to a control locality.

## INTRODUCTION

In many countries there continues to be intense public interest in the trading hours of alcohol outlets. Encouragingly, local, state and national governments appear increasingly interested in the application of research evidence to the regulation of the liquor trade. Given the demand for evidence, there is surprisingly little research literature on the effects of changes in trading hours. In the two major reviews of empirical evidence on alcohol policy in recent decades [1,2], there are just a few short paragraphs on the role of trading hours.

The tendency in the post-war years in many countries has been to liberalize alcohol control policies [1], such that what evidence exists pertains mainly to the effects of later closing (i.e. liberalization of trade), with only a few studies of the effects of earlier closing (i.e. restriction of trade). A recent narrative review by Stockwell & Chikritzhs [3] of the effects of changes in trading hours examined 14 studies employing pre–post measurement and control sites, of which 13 were liberalization studies. In general, increasing trading hours was reported to be associated with a higher incidence of alcohol-related harm [3].

Four further studies, three of which were not covered in the review by Stockwell & Chikritzhs (i.e. [4–7]) examined the effects of regulations requiring earlier closing. Consistent with the liberalization studies referred to above, the typical finding was that earlier closing was associated with less alcohol-related harm. It should be noted, however, that compared with the liberalization studies, these restriction studies generalize less well to the circumstances faced by most liquor licensing policy makers which, typically, do not include management of national border crossings or remote indigenous communities.

The present investigation arose from a regulatory change applied in Newcastle, New South Wales (NSW), Australia's seventh largest city (population 530 000). Licensed premises with late trading licences in the central business district (CBD) of Newcastle had been shown to have a high incidence of assault [8] and, more generally, intoxication in licensed premises in NSW was reported to be commonplace despite a law proscribing admission or service of intoxicated individuals [9].

In NSW, alcohol outlet licensing is managed by the State Government's Office of Liquor Gaming and Racing (known as the Liquor Administration Board until 30 June 2008). In 2007, formal complaints about violence, damage to property and disorderly behaviour arising from service to intoxication in the Newcastle CBD were made by the NSW Police and members of the community. As a result, in 2008 the Liquor Administration Board restricted opening hours of 14 pubs in the CBD from 5 a.m. to 3 a.m., with a 1 a.m. lockout, effective from 21 March 2008. Under the lockout conditions patrons could continue to drink alcohol on the premises until 3 a.m. but no new patrons could be admitted after 1 a.m., thus it is also known as a ‘one-way door’ policy.

The pubs mounted a legal challenge to the ruling and as a consequence of an out-of-court bargain with the NSW police on 29 July 2008, the restriction was relaxed to 3.30 a.m. closing with a 1.30 a.m. lockout. We sought to test the hypothesis that this intervention would reduce the incidence of assault in the Newcastle CBD. In addition, we sought to determine whether there was any displacement in assault incidence from the CBD to the nearby control area.

## METHODS

### Design

We adopted a non-equivalent control group design [10] in which the CBD was the intervention area and a nearby area with similar characteristics served as the control area. Ideally, one would have several control areas, all affected identically by determinants of drinking and other assault risk factors, e.g. by macro-economic conditions and transport variables. They would consist of the same demographic mix of patrons, the same types of outlets, be beyond convenient walking distance from the intervention area and be smaller than the intervention area, so that displacement from the intervention area could be detected readily.

### Study sites

**Figure 1:**
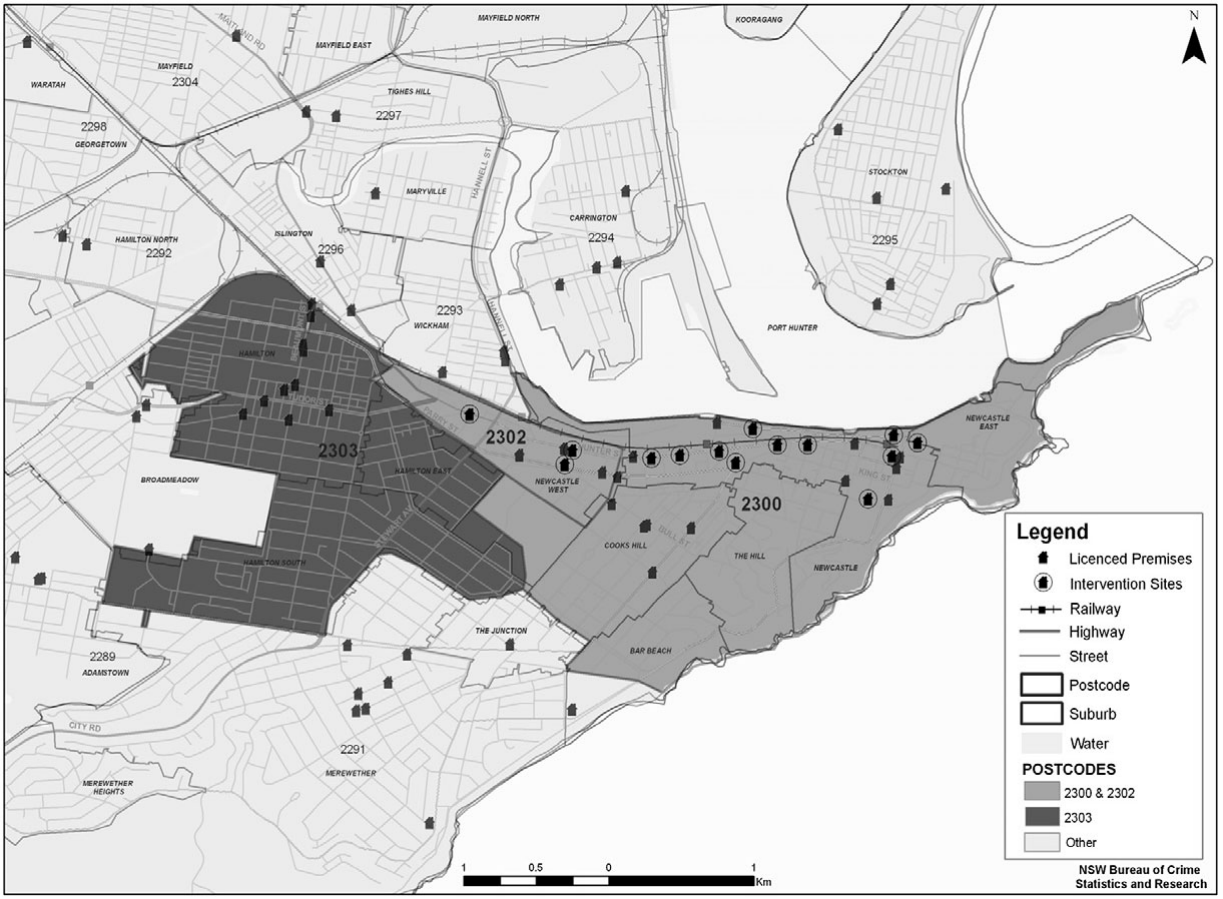
Location of study sites: central business district (CBD) (postcodes 2300 and 2302) and Hamilton (postcode 2303)

Figure 1 shows the location and boundaries of the CBD 2300 and 2302 postcode areas (intervention) and the Hamilton 2303 postcode area (control). Hamilton was selected as a control area because, like the CBD, it is considered an entertainment precinct and includes several late trading pubs of similar character to those in the CBD, and because closing times were not curtailed. Critically, it would be subject to similar economic, transport and climatic conditions, all of which are known to affect drinking behaviour in public locations. As shall be seen, the perpetrators and victims of assault in Hamilton are approximately 5 years older than those in the CBD, and the area occupied by pubs is considerably smaller than that in the CBD. In summary, Hamilton has many, but not all, of the features of an ideal control site and there are no other entertainment precincts in the Newcastle region suitable for comparison.

### The intervention

In addition to the changes in closing hours described above, licensees were required to adopt a plan of management; were subject to compliance audits; had to have a dedicated responsible service of alcohol officer from 11 p.m. until closing; could not serve shots after 10 p.m.; had to cease selling alcohol 30 minutes prior to closing; could not permit stockpiling of drinks; had to adopt shared radio procedures; and all staff had to be notified of the conditions. Importantly, pubs in Hamilton, the control area, reportedly began to adopt most elements of the intervention voluntarily from November 2008. Later reports cast doubt on the degree of compliance with the voluntary restrictions in Hamilton [11].

### Case definition

Cases were non-domestic violence incidents that were reported to or detected by police. Assault incidents included common assault, actual or grievous bodily harm, assault of police or shooting with intent other than to murder, as defined under the NSW Crimes Act 1900, and irrespective of whether or not there was a subsequent charge or conviction. Cases were limited to those occurring between 10 p.m. and 6 a.m. within either the CBD postcode areas or the Hamilton postcode area. Such incidents could include any number of people who were recorded as either a person of interest (i.e. a possible perpetrator) or victim. It should be noted that the analysis of the effect of the restriction in closing times was based upon the count of incidents, not of individuals.

The intervention took effect on 21 March 2008. At the time of the study, post-test data were available to 30 September 2009. A relatively stable period in assault incidence before the law change, namely April 2001–March 2008 (28 quarters), was chosen for comparison with the post-intervention period of April 2008–September 2009 (six quarters).

### Analysis

We used negative binomial regression to model the number of assaults per month in the before and after periods. The negative binomial model included a variable to indicate the periods before and after the intervention and a variable to indicate the area in which the assault occurred. The difference in the change in the number of assaults across the intervention period between the two areas was tested using an interaction term between the before and after variable and the area variable. The exponent of the coefficient of the interaction term from this model, that is the incidence rate ratio (IRR), is an estimate of the relative difference in the percentage change in the number of assaults in the CBD compared with Hamilton [12].

Additionally, a variable for the time (in months) from the start of the study was added to the model to adjust for any secular trend in assaults that may have occurred over the study period, and a categorical variable for month of the year was added to adjust for any seasonal variation. The results presented in the tables within this paper are from a model that does not adjust for serial autocorrelation. We did, however, fit the same model into a generalized estimating equation (GEE) framework, which allowed us to include an autoregressive term to adjust for autocorrelation within cluster, but there are concerns about the standard errors of these models being unduly small when the number of clusters is small [13]. STATA's implementation of a GEE allows the use of bootstrapping and the effect sizes and 95% confidence intervals (CIs) estimated from the bootstrap models are presented in the text. In addition, we tested the robustness of the results using a traditional time–series approach, i.e. by fitting an autoregressive integrated moving average (ARIMA) model separately for the time–series within each area. The results of these two models were entirely consistent with the findings from the negative binomial regression models and GEEs and they are not reported here.

To examine any temporal shift in the number of assaults we refitted the above models restricting the data to the two separate time periods of 10 p.m.–2.59 a.m. and 3 a.m.–6 a.m. The analyses were repeated for incidents occurring between 6 p.m. and 9.59 p.m. to test for the possibility that patrons shifted their drinking (and therefore assaults) to a much earlier period.

The number of events that would have occurred in the CBD had the change in closing times not taken place was estimated by multiplying the average number of events observed per quarter in the CBD prior to the intervention by the IRR across the intervention period in Hamilton. The number of events prevented by the intervention was estimated by subtracting the number of events that actually occurred in Newcastle from the number estimated to have occurred if the change in closing times had not taken place. Chi-square tests were used to examine differences in the percentage of assaults between 10 p.m. and 6 a.m. that occurred after 3 a.m. within each area.

It is possible that as a consequence of being under regulatory scrutiny, licensees in the CBD under-reported assaults to police after the intervention was initiated to a greater extent than beforehand. We therefore undertook a manual review of reports to police according to their source, before and after the intervention commenced in the CBD and Hamilton, by way of assessing this potential threat to the validity of findings. Given the labour-intensiveness of the manual search, this could be conducted for only one quarter before the change (October–December 2007) and the corresponding quarter in the following year (October–December 2008).

## RESULTS

### Assault incidence in the study sites before and after the intervention

**Figure 2:**
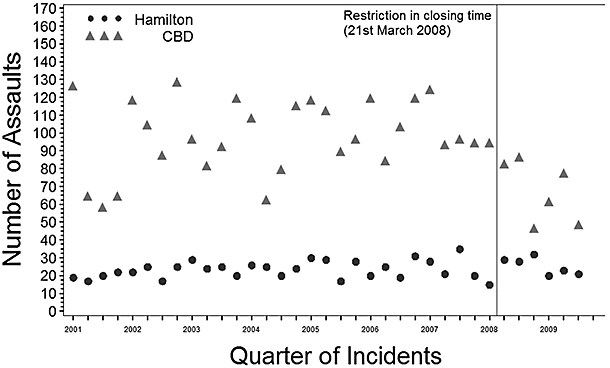
Assaults per quarter, January 2001–September 2009, in central business district (CBD) (intervention area) and Hamilton (control area)

Figure 2 shows the number of assaults in the January–March, April–June, July–September and October–December quarters in the period January 2001 to September 2009, in the CBD and Hamilton. The figure suggests a gradual increase in assault incidence in the Hamilton area. The series appears more volatile in the CBD, although it should be noted that this is due mainly to scaling effects. There was a dramatic reduction in assaults in the final quarter of 2008 followed by an increase in the first two quarters of 2009 and a decrease in the third quarter. Overall, counts for the last four quarters of the series were well below the range of values expected in the absence of an intervention.

### Demographic details of assault perpetrators and victims

Table 1 presents the age and gender distributions of persons of interest (who include suspected assailants) and assault victims in each area before and after the intervention. It should be noted that in contrast to the analyses concerning the effects of the change in closing times, which are incident-based, the summary presented in Table 1 is person-based. This is due to the fact that for any particular assault investigated by police, there could be several persons of interest and/or victims. Table 1 shows that the perpetrators and victims of assault are overwhelmingly young men. Perpetrators and victims of assault were, on average, 5 years older in Hamilton than in the CBD.

**Table 1:** Gender and age distributions of people involved in assaults in the study areas, before and after the change in closing time

	CBD		Hamilton	
		
	Males	Females	Males	Females
Person of interest				
Before^a^	1541 (82%)	340 (18%)	381 (83%)	80 (17%)
After	209 (82%)	46 (18%)	79 (80%)	20 (20%)
Mean age (SD)	23.8 (7.4)	21.5 (6.7)	28.7 (9.4)	26.1 (7.8)
Victim				
Before	2705 (81%)	619 (19%)	644 (83%)	131 (17%)
After	377 (76%)	118 (24%)	141 (77%)	41 (23%)
Mean age (SD)	25.6 (8.3)	23.7 (7.6)	30.5 (9.8)	29.3 (9.7)

a Before: January 2001–March 2008; after: April 2008–September 2009. CBD: central business district; SD: standard deviation.

### Test of primary hypothesis

Table 2 summarizes the primary results. It shows that there was a 34% reduction in assault incidence in the intervention area and a non-significant increase of 2% in the control area in the same period. The relative effect, i.e. the effect of the intervention adjusting for the assault incidence in Hamilton, is given by the ratio of the incidence rate ratios in the two study sites, i.e. a 37% relative reduction [(1–0.63) × 100], which equates to 33 assaults prevented per quarter [(99.0 × 1.02)−67.7]. Analysed with the GEE bootstrapped models, the effect estimate was identical (IRR: 0.63) to that in the negative binomial regression model, albeit with a wider confidence interval (95% CI: 0.40–0.99).

**Table 2:** Assaults per quarter before and after the change in closing time

	Before^a^ n	After^a^ n	Before-to-after incidence rate ratio^b^ (95% CI)	Relative before-to-after incidence rate ratio^b^ (95% CI)	P
CBD (intervention area)	99.0	67.7	0.66 (0.55–0.80)	0.63 (0.49, 0.81)	0.0003^c^
Hamilton (control area)	23.4	25.5	1.02 (0.79–1.31)	1.00 Reference	–

a Before: January 2001–March 2008; after: April 2008–September 2009.

b Incidence rate ratios are adjusted to take into account the variation by month of the year (seasonal effect) and time since January 2001 (secular trend) and therefore they are not necessarily the same as those estimated by division of crude numbers within the table.

c For area × time interaction term in negative binomial regression model.

CBD: central business district; CI: confidence interval.

When the data were analysed separately by time of incident, effect estimates were markedly larger for assaults occurring between 3 a.m. and 6 a.m. (67% relative reduction; IRR: 0.33, 95% CI: 0.19–0.56) than for those occurring between 10 p.m. and 2.59 a.m. (26% relative reduction; IRR: 0.74, 95% CI: 0.56–0.89). For the earlier period (6 p.m.–9.59 p.m.) there was a non-significant increase in assault incidence in the CBD (from 15 to 17.5 assaults per quarter, RR: 1.17, CI 0.9–1.5), and no change in Hamilton (9.3 per quarter before and after the restriction, RR: 1.0; CI 0.7–1.4).

### Test of secondary hypothesis

**Figure 3:**
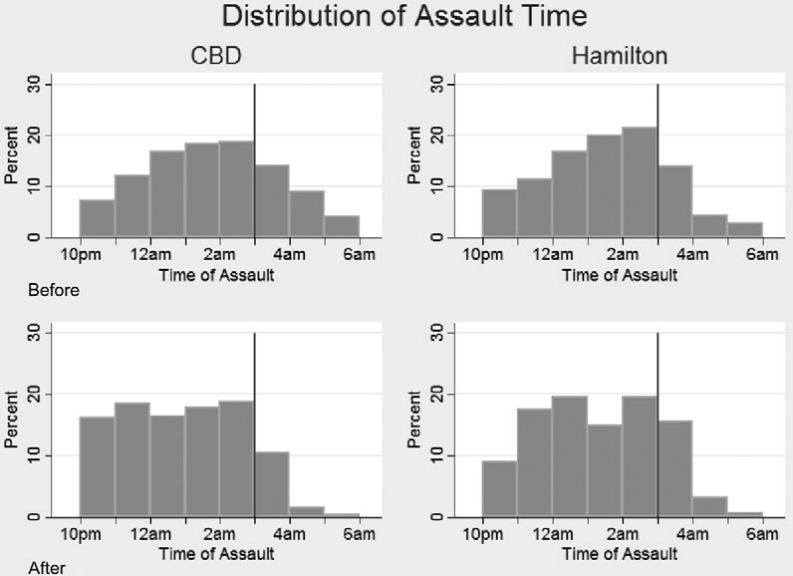
Distributions of assaults by time in central business district (CBD) (intervention area) and Hamilton (control area) before and after the change in closing time

In the CBD before the intervention 27% of assaults occurred after 3 a.m. This decreased to 12% after the intervention (*P* < 0.0001). In Hamilton, corresponding figures were 21% and 20% (*P* = 0.65). Figure 3 illustrates this finding, suggesting that the intervention effect shown in Table 2 occurred via the anticipated mechanism of reducing the overall number of assaults in the CBD without causing displacement to nearby Hamilton after 3 a.m. or 3.30 a.m. closing.

### Examination of potential selection bias

Table 3 presents the number and proportion of assaults recorded in the CBD and Hamilton, by person reporting the assault and year (October–December of 2007 versus October–December of 2008). Pub staff reported fewer than 10% of the incidents in the data set, and the change in the number of events reported over time in the CBD (9.9–7.7%) was non-significant. No support was found for the hypothesis that the reporting practices of licensees could explain the differences evident in Table 2.

**Table 3:** Number and proportion of assaults recorded in central business district (CBD) and Hamilton, by person reporting the assault and year

	Reported/detected by				
	
	Pub staff	Police	Victim	Other	Unclear
Location/time period	n (%)	n (%)	n (%)	n (%)	n (%)
CBD					
October–December 2007	13 (9.9)	16 (12.2)	51 (38.9)	40 (30.5)	11 (8.4)
October–December 2008	7 (7.7)	6 (6.6)	35 (38.5)	35 (38.5)	8 (8.8)
χ^2^_4_ = 3.0, *P* = 0.554					
Hamilton					
October–December 2007	4 (8.2)	1 (2.0)	19 (38.8)	20 (40.8)	5 (10.2)
October–December 2008	1 (1.7)	6 (10.3)	24 (41.4)	20 (34.5)	7 (12.1)
χ^2^_4_ = 5.6^a^, *P* = 0.234					

a Note:counts of <5 in some cells affect the reliability of the χ^2^ statistic.

## DISCUSSION

The principal finding is consistent with the primary hypothesis, i.e. the restriction in closing time appears to have produced a reduction in assault incidence against a backdrop of a stable trend in the control area. This was despite a watering-down of the original restriction (from 3 a.m. to 3.30 a.m. 4 months in) and possible contamination in the form of voluntary adoption of some intervention elements in the control site. There does not appear to have been geographic displacement to Hamilton, i.e. an increase in assaults as a consequence of patrons either moving to Hamilton from the CBD after 3.30 a.m. closing or choosing to frequent Hamilton pubs instead of those in the CBD. Displacement to other areas of Newcastle cannot be ruled out; however, it should be noted that there are no other entertainment precincts in the city with clusters of late trading pubs.

Notably, significant reductions in assault rates were evident only in the third quarter after the law change. A lag is plausible—it may have taken time for patrons' patterns of going out drinking to change in response to the new closing times. It should also be noted that in the first two quarters after the restriction took effect, assaults increased in the control area relative to the preceding two quarters.

Strengths of the study include the use of a control site which confers significantly greater capacity for a valid causal inference over the one-group pre-test–post-test design [10]. For example, this design reduces the likelihood that macroeconomic factors, some of which are known to affect drinking behaviour [2], biased the analysis. In the period studied there was a global economic crisis and dramatic changes in the price of petrol, both of which will have affected how much money people could spend on going out and purchasing alcohol, and therefore may have reduced the total exposure to the risk of assault. These effects are unlikely to have occurred differentially in the CBD and Hamilton and therefore the effect estimate should not have been biased.

A priori limitations of the study include possible differences in police activity and pub staff reporting of assaults in the two areas before and after the restriction. The former is an example of a service delivery variable potentially confounding valid causal inference [14]. If, as a consequence of the intervention, more police were temporarily put onto the street in the CBD and/or they became more zealous than usual in apprehending people for assault, the detection rate may have been inflated artificially. This will have resulted in underestimation of the intervention effect. It is difficult to imagine a plausible scenario in which this bias could operate in favour of the study hypothesis; however, in the absence of independent data on policing levels it is impossible to do more than speculate.

A more plausible threat to the validity of the effect estimate might be that in the wake of prominent adverse publicity about assaults in and around licensed premises, pub owners advised their staff to avoid calling the police in the event that a patron committed an assault on or near the premises. Such a practice would have artificially deflated police counts of assault incidence upon which our estimates depended. If this occurred to a greater extent in the CBD than in the Hamilton area, and more so after the intervention than before, the intervention effect could have been overestimated; however, our analysis of the source of assault records showed that this did not occur. Given that fewer than 10% of assault reports originated with licensed premises, it would have been impossible for this to explain the observed changes in assault incidence even if such a practice had been adopted completely.

While the above suggests that the observed time × area interaction (i.e. the intervention effect) is not artefactual, it remains possible that the effects are due, wholly or in part, to factors other than the restriction in closing times. At the time of the intervention, pubs were subject to adverse publicity from media reports in March 2008 [15] of a ‘top 100 list’ obtained from the NSW Bureau of Crime Statistics and Research. The report ranked the 100 pubs in NSW with the largest number of assaults occurring on the premises. Notably, five of those pubs were in the CBD (36% of all the pubs subject to the intervention and 17% of all the pubs in the CBD) and three were in Hamilton (30% of all the pubs in the control area). It is likely that as a consequence of the publicity, pubs modified their service and security practices and this may have reduced assault rates independently of the restriction in closing times. However, given that ‘top 100’ pubs were present in both the intervention and control sites, any such effect is unlikely to have biased the closing time effect estimate.

Other changes that occurred during the period covered in this evaluation include the introduction of a new Liquor Act, which came into effect on 1 July 2008, and the announcement by the NSW Premier of ‘Top 48’ legislation in October 2008, which imposed various restrictions on the service practices of pubs with the worst assault records. As above, the inference concerning the effect of restrictions in closing times is protected by the inclusion of a control site subject to the same conditions as those in place in the intervention site.

The voluntary adoption of aspects of the intervention by some pubs in the control area from late 2008 creates the possibility that the effect estimate has been biased towards the null. Figure 2 shows that the assault incidence rates in Hamilton were lower in 2009 than in 2008, so it is possible that the voluntary measures had a small protective effect. If so, the true effect of the restriction placed on pubs in the CBD area would be greater than that estimated here.

There may be benefit in analysing outcomes that are less susceptible to selection biases. While emergency department admissions for assault are an obvious candidate, the location of the assault incident is not recorded routinely in the medical record, making it impossible to distinguish between incidents in the CBD and other areas. Ambulance attendances for assault appear to be a possibility as long as the location of the patient at the time of the assault can be ascertained, which is currently being investigated.

The findings are consistent with the small literature on restriction studies and therefore with the broader availability hypothesis; namely, that increasing the physical and/or economic availability of alcohol increases consumption and therefore alcohol-related harm [1]. It should be noted that in practice it is rare for physical availability to increase without also increasing the promotion of alcohol (e.g. in ‘happy hour’ advertising, at point of sale, etc.), i.e. the supposed mechanism of action is not only supply-side, but also involves stimulating demand for alcohol.

There are also factors not related directly to alcohol consumption that affect the incidence of assault, e.g. overcrowding, social deprivation and patron mix [16,17]. By restricting closing times, the intervention may have reduced the number of people coming into the CBD and thereby reduced the likelihood of aggressive interactions between patrons within, outside and travelling between licensed premises.

The intervention appears to have reduced assaults after 3 a.m. dramatically (by two-thirds), even though the latest permissible closing time for 14 of the 18 post-intervention months was 3.30 a.m. The relative contribution of there being possibly fewer patrons in the CBD after 3 a.m. than previously, and that those who were present were less intoxicated, is unknown. That there was an intervention effect (a 26% relative reduction) between 10 p.m. and 2.59 a.m. suggests that reduced exposure (i.e. fewer people visiting the CBD area) may explain at least part of the observed reduction in assaults later on. In addition, it is possible that aspects of the intervention other than the restriction in closing times affected patron behaviour via modification of service and other management practices.

There are several reasons to be cautious about these results: (1) the possibility that the two areas are not sufficiently comparable to form a valid counterfactual to the intervention (e.g. assault perpetrators in Hamilton were 5 years older than those in the CBD); (2) that an effect was only seen after a two-quarter lag; and (3) the presence of an effect (albeit smaller) at earlier as well as later times. In relation to the first point, it should be noted that in this particular case the result of the conditional analysis (i.e. of the change in the CBD versus that in Hamilton) was not sensitive to what occurred in the control site because assault incidence was stable in the period in question. With regard to the third point, it should be noted that changes in trading hours shown in previous studies to affect rates of assault and other harms (see [3] for a review) occurred largely in the absence of the kinds of strategies introduced in the CBD (e.g. the ban on shots after 10 p.m.). These findings, and the lack of evidence one way or the other on the effects of the other strategies implemented in the CBD, lend support to reduced exposure as an explanation for the reduction in assaults observed between 10 p.m. and 3 a.m.

The lack of data on patron travel behaviour (e.g. counts of people moving into and out of the area on Saturday nights by various modes of transport) and drinking behaviour (e.g. breath alcohol levels measured at sentinel locations at specified times, or pub alcohol sales data) makes it impossible to determine whether the intervention worked via the assumed mechanisms. It underlines the importance of designing evaluations in anticipation of important policy changes such as that examined here, which would require government to adopt a more active role as a contributor to the development of research evidence rather than being merely a consumer of it [18].

In addition to examining other sources of data (e.g. ambulance attendances) in relation to the Newcastle intervention, further research is required to examine the effects of lockouts. These are now used widely but there is little or no evidence concerning their effectiveness. In the meantime, licensing authorities presented with similar assault and disorder problems may be emboldened by these findings and should be encouraged to implement similar restrictions with suitable evaluation.
